# A Novel Integrated Score Index of Echocardiographic Indices for the Evaluation of Left Ventricular Diastolic Function

**DOI:** 10.1371/journal.pone.0142175

**Published:** 2015-11-10

**Authors:** Sheng-Nan Chang, Jimmy Jyh-Ming Juang, Chia-Ti Tsai, Jiing-Tang Ko, Wen-Pin Lien

**Affiliations:** 1 National Taiwan University College of Medicine, Graduate Institute of Clinical Medicine, Taipei, Taiwan; 2 Division of Cardiology, Department of Internal Medicine, National Taiwan University Hospital Yun-Lin Branch, Dou-Liu City, Taiwan; 3 Cardiovascular Center, Department of Internal Medicine, National Taiwan University Hospital, Taipei, Taiwan; 4 Nuliv Wellness Clinic, Taipei, Taiwan; University Heart Center, GERMANY

## Abstract

**Background:**

We propose a novel integrated score index, which could be used to quantify and grade left ventricular (LV) diastolic function.

**Methods:**

We enrolled 629 participants [393 healthy subjects, 145 with hypertension (HTN), 24 with hypertrophic cardiomyopathy (HCM), and 67 with coronary artery disease (CAD)]. This score index was with a score of 1 for an E/A ratio < 1, a score of 1 for a septal e’/a’ ratio ≤ 0.8, a score of 2 for a lateral e’/a’ ratio ≤ 1, a score of 2 for a septal E/e’ ratio ≥10–15, a score of 3 for a lateral E/e’ ratio ≥8–15, and a score of 1 for a deceleration time >240 ms. The sum of each score was considered as the final value in this scoring method (either a septal or a lateral E/e’ ratio > 15 was given a total score of 10, regardless of the other measurements).

**Results:**

After analysis, the AUROC of this integrated score index for predicting any diastolic dysfunction (discriminated by the American Society of Echocardiography guidelines) was 0.962, and the AUROC of the method from the logistic regression was 0.970. The mean values of the score index for the groups were 3.81 ± 0.12 in healthy, 6.48 ± 0.19 in HTN, 7.35 ± 0.46 in HCM, and 6.62 ± 0.29 in CAD. Using the score index, the healthy subjects obtained lower scores compared with those of HTN (p = 0.00), HCM (p = 0.00), and CAD (p = 0.00). Therefore, this score index could discriminate patients with diseases with impaired diastolic function from the healthy subjects when the total sum of the score was equal to or greater than 4.

**Conclusions:**

If the presently used methods cannot allow the clear diagnosis of LV diastolic dysfunction, this integrated score index might be helpful for discriminating diseases with impaired diastolic function.

## Introduction

Recent reports have suggested that half of the patients diagnosed with heart failure have normal or near normal global left ventricular ejection fraction (LVEF).[1–5] These patients are referred to as having heart failure with normal ejection fraction or as having diastolic heart failure (DHF). Even in Asian countries, the prevalence of DHF is increasing, perhaps due to the rapidly aging population, to diseases associated with metabolic components such as metabolic syndrome or westernized lifestyle including dietary patterns, to decreasing physical activity, and to obesity.[6–8] Accordingly, the importance of quantitating diastolic function and diagnosing DHF is increasingly recognized. The standard method for demonstrating LV diastolic dysfunction is cardiac catheterization, which can produce pressure-volume curves during isovolumic relaxation.[9] However, this measurement is imperfect because routine invasive catheterization is not feasible. Two-dimensional (2-D) echocardiography and tissue Doppler imaging (TDI) have emerged as the preferred non-invasive modalities by which diastolic function is assessed for clinical or research purposes.[10–13] However, the clinical diagnosis of DHF remains challenging, and no single echocardiographic parameter appears to be sufficiently accurate and reproducible as a sole measurement for the diagnosis of LV diastolic function. Even with the current guidelines published by the American Society of Echocardiography [14] and European Society of Cardiology [15] for the echocardiographic assessments of diastolic function, the diagnostic process for incorporating the individual data is complex. Moreover, the diastolic function is only classified by the grades of normal, I, II, and III rather than by continuous scores according to the current diagnostic suggestions.[14] Because the early identification of high-risk subjects with diastolic dysfunction remains mandatory for preventing heart failure and improving prognosis, we designed this novel integrated score index to assess cardiac diastolic function. The application of this score index in patients with hypertension (HTN), hypertrophic cardiomyopathy (HCM), and coronary artery disease (CAD) could also help us find the presence of sub-clinically diastolic dysfunction and risk groups for heart failure, which might be helpful for further therapeutic adjustment in clinical applications.

## Materials and Methods

### Participants

This was a single-center study, which included consecutive patients referred for transthoracic echocardiography with sufficient 2-D image qualities. Initially, from the year 2011 to 2013, 526 consecutive subjects were enrolled in this study. The inclusion criteria were healthy subjects with normal systolic LV function (left ventricular ejection fraction, LVEF ≥ 50%), as assessed by 2-D echocardiography. All subjects underwent 2-D echocardiography and TDI examinations. The exclusion criteria were coronary artery disease, rheumatic valvular disease, severe mitral or aortic regurgitation, significant aortic stenosis (peak valvular systolic gradient > 40 mmHg), congenital heart disease, cardiomyopathy, arrhythmias, a history of congestive heart failure or symptoms and/or abnormal LVEF < 50%, hypertension, diabetes mellitus, chronic obstructive pulmonary disease, thyroid disease, cerebrovascular accidents, malignancy, body mass index ≥ 30 kg/m^2^, subjects with a poor acoustic window or with echocardiographic evidence of heavy mitral annulus calcification, hemoglobin < 13 g/dl, albumin < 3.2 g/dl, or serum creatinine ≥1.4 mg/dl. The criteria for hypertension were as follows: 1) for subjects < 70 years old, systolic pressure > 150 mmHg and diastolic pressure > 90 mmHg, and 2) for subjects ≥ 70 years old, systolic pressure > 170 mmHg and diastolic pressure > 90 mmHg. Finally, only 393 healthy subjects were enrolled for further analysis. The baseline information, the characteristics, and the changes in cardiac structures of the 393 healthy participants were presented according to the different age categories in S1 File

For the clinical use of this integrated score index, patients who were likely to have impaired diastolic function with HTN (145 cases), HCM (24 cases), and CAD (67 cases) were enrolled as the compared groups. The baseline information and characteristics of these patients were presented in S2 File. The medications of the healthy subjects and the patients with possibly impaired diastolic function were presented in S3 File.

This study was approved by the ethics committee and institutional review board (IRB) on human research of the Medical Research Department of National Taiwan University Hospital, Taipei, Taiwan. All subjects provided informed consent before participating in the study. The participants provided their written informed consent to participate in this study.

### Echocardiographic studies based on 2-D and tissue Doppler measures

For all studies, 2-D echocardiography and TDI were performed using the Doppler echo imaging system (GE Company, Vivid S5) equipped with a 1.7/3.4-MHz imaging transducer according to recommendations of the American Society of Echocardiography.[14] The trans-mitral Doppler flow velocity was measured using a 5-mm sample volume placed at the tips of the mitral leaflets in passive end expiration. A standardized loop of 10 cardiac cycles was downloaded to a computer for off-line analysis of the early filling (E-wave) and late filling (A-wave) phases.[14] Tissue Doppler velocities were then acquired at the level of the septal and lateral mitral annulus. The early diastolic e’ velocity and a’ velocity at the septal and lateral annular site were measured, and the e’/a’ and the E/e’ ratio were calculated.[14] To obtain accurate data while performing Doppler measurements, the septal and lateral walls were highlighted in the apical 4-chamber view. Using pulse-wave Doppler, a sample volume of 4.0 mm was placed at the septal side of the mitral annulus. This process was then repeated for the lateral side of the mitral annulus. Each measurement of septal or lateral e’ was repeated nine times. Then, the values were averaged to obtain a mean septal or lateral e’ value. Baseline clinical information, biochemical data, and medical histories were collected.

To evaluate the LV diastolic function of healthy subjects, we proposed an integrated score index of echocardiography. This score index was based on the sum of measurements from echocardiography, including the 2-D echocardiography and the TDI at the mitral annulus. From the diagnostic algorithm, a scheme for grading diastolic dysfunction, and the normal values for Doppler-derived diastolic measurements suggested by the American Society of Echocardiography, [14] we chose measurements of the E/A ratio, septal e’/a’ ratio, lateral e’/a’ ratio, and deceleration time as our scoring categories.[13] We also used the measurement of E/e’ in this integrated score index based on the suggestion of the European Society of Cardiology.[12]

The cut points of each scoring category were summarized from the normal values of Doppler-derived diastolic measurements, according to the current recommendations.[5, 14, 15] Our criteria for this integrated score index were as follows: a score of 1 for an E/A ratio < 1, a score of 1 for a septal e’/a’ ratio ≤ 0.8, a score of 2 for a lateral e’/a’ ratio ≤ 1, a score of 2 for a septal E/e’ ratio ≥10–15, a score of 3 for a lateral E/e’ ratio ≥8–15, and a score of 1 for a deceleration time >240 ms (Table 1). The sum of each score was considered as the final value in this score index. Either a septal or a lateral E/e’ ratio > 15 was given a total score of 10, regardless of the other measurements. Additionally, we divided the healthy subjects into five age groups (group 1: aged ≤ 29 years, group 2: aged 30–49 years, group 3: aged 50–69 years, group 4: aged 70–89 years, and group 5: aged ≥ 90 years). With the help of the score index, we then compared the diastolic function of different age groups. By collecting basic data from the different age groups of healthy subjects, we were able to evaluate the diastolic function score between different age groups. This enabled identification of the association between aging and diastolic dysfunction. We also determined the diastolic function score of each participant by using this integrated score index. Based on these scores, we grouped the diastolic function score as scores of 0–2, scores of 3–5, and scores of 6–10. The frequencies of the study population with different scores were compared with each other among the individual age groups.

**Table 1:** An integrated score for evaluating left ventricular diastolic function. MV = mitral valve, E = early diastolic flow velocity, A = late diastolic flow velocity, e’ = mitral annulus early diastolic velocity, a’ = mitral annulus late diastolic velocity, DT = deceleration time

	Value	Score
MV E/A ratio	< 1	1
MV e'/a' ratio (septal)	≤ 0.8	1
MV e'/a' ratio (lateral)	≤ 1.0	2
MV E/e' ratio (septal)	≥ 10–15	2
MV E/e' ratio (lateral)	≥ 8–15	3
MV DT (ms)	> 240	1

### Statistical analysis

Continuous data were expressed as the mean ± standard deviation (SD) and were compared using a nonparametric trend test. Incidence data were expressed as a proportion and were compared with the Chi-square test or Fisher’s exact test. Differences between the continuous data of groups were determined by performing one-way analysis of variance (ANOVA) followed by the Bonferroni post hoc test for multiple paired comparisons. The statistical power was calculated in a pre-specified manner, with an alpha error of five percent capable of revealing significant differences with a statistical power > 90% for deformation data. Logistic regression analysis was performed to estimate regression parameters.

The study population was further subdivided into diastolic dysfunction or normal cardiac function groups according to the current guidelines of the American Society of Echocardiography [14]. Receiver operating characteristic (ROC) curve analysis was used with an optimal cut-off generated from the largest sensitivity and specificity summation for diastolic dysfunction and with discriminations of the area under the ROC curve (AUROC and its 95% confidence interval (95% CI)) in clinical stratification using C-statistics. A two-tailed *P-*value < 0.05 was considered statistically significant. Statistical analyses were performed using STATA software for Windows, version 9.0 (Stata Corporation, College Station, Texas).

## Results

### Baseline characteristics of the healthy population

The data obtained from the different age groups and gender were finally analyzed. Subjects in the elderly population groups tended to show higher systolic blood pressure (SBP): Gr 3 vs. Gr 4 + 5: 125.8 mmHg vs. 145.0 mmHg, *P* < 0.01. The pressure gradients of the mitral valve (MV), tricuspid valve (TV), and aortic valve (AV) were higher in the group of elderly subjects: Gr 3 vs. Gr 4 + 5: 2.68 mmHg vs. 4.25 mmHg, *P* < 0.01; 20.24 mmHg vs. 24.97 mmHg, *P* < 0.01; 5.93 mmHg vs. 10.01 mmHg, *P* < 0.01, respectively. The aortic root (AO) and posterior wall were also higher in the group of elderly subjects: Gr 3 vs. Gr 4 + 5: 3.17 cm vs. 3.36 cm, *P* < 0.01 and 0.82 cm vs. 0.88 cm, *P* < 0.01, respectively. Between the elderly and the younger groups, no significant association of other parameters such as body mass index (BMI), LVEF, diastolic blood pressure (DBP), left atrial dimension (LA), left ventricular end-diastolic dimension (LVEDD), and left ventricular end-systolic dimension (LVESD) was noted.

Within the different age groups, there was no consistent gender difference in the indices of blood pressure (systolic and diastolic), cardiac structures, or valvular pressure gradients. However, male participants showed greater LVEDD, LVESD, and AO than the female participants both in Gr 3 (male vs. female: 4.67 cm vs. 4.43 cm, *P* < 0.01; 2.55 cm vs. 2.30 cm, *P* < 0.01; 3.47 cm vs. 2.96 cm, *P* < 0.01, respectively) and in Gr 4 + 5 (male vs. female: 4.74 cm vs. 4.42 cm, *P* < 0.01; 2.59 cm vs. 2.26 cm, *P* < 0.01; 3.60 cm vs. 3.21 cm, *P* < 0.01, respectively).

### Comparison and correlation between age and diastolic function in healthy population

**Fig 1:**
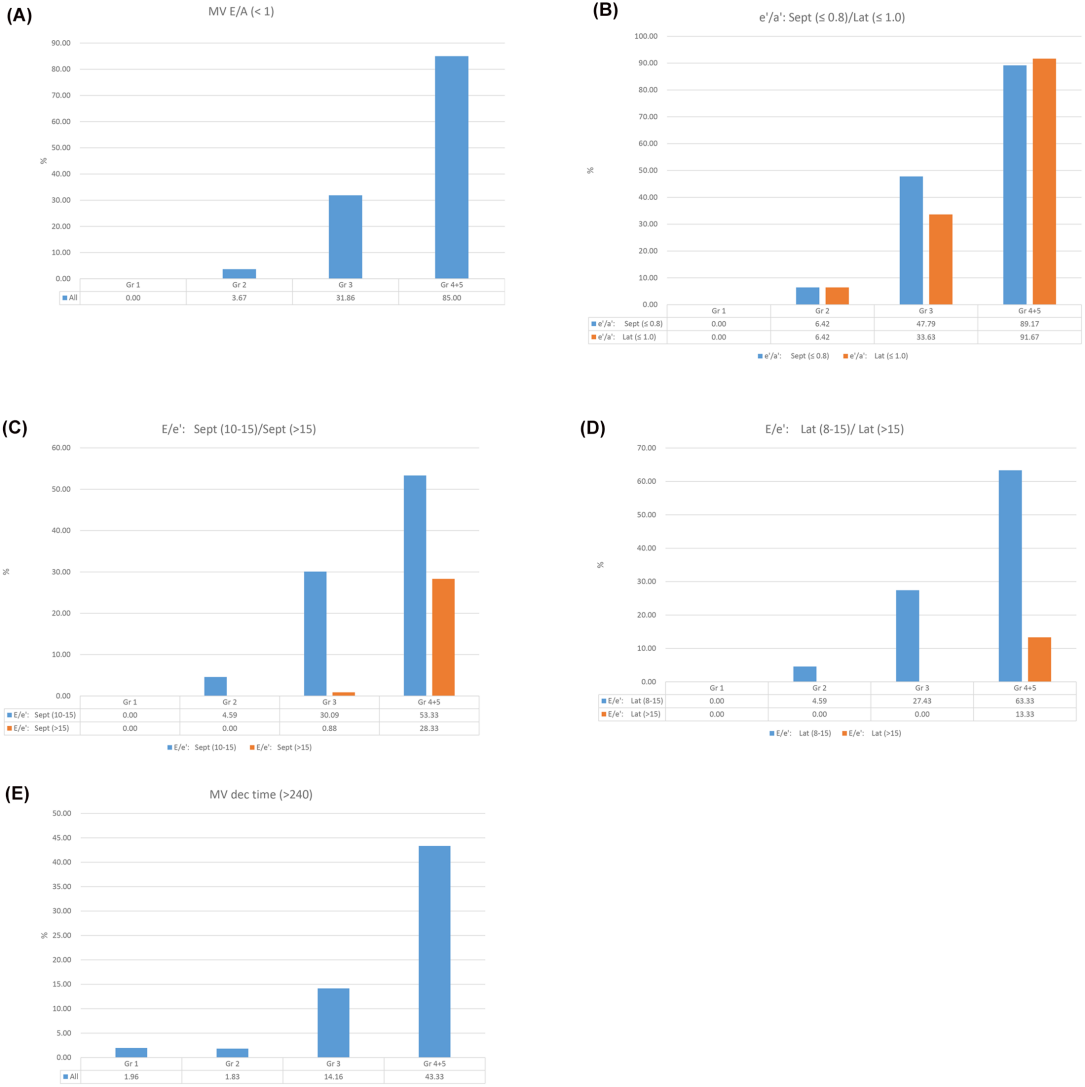
Left ventricular diastolic function in the healthy population, as assessed with individual indices. (A) MV E/A <1. (B) e’/a’ septal ≤0.8, lateral ≤1.0. (C) E/e’ septal 10–15; septal >15. (D) E/e’ lateral 8–15, lateral >15. (E) MV deceleration time >240.

We evaluated the LV diastolic function in the healthy population by using our integrated score index. The final mean value from the summation of each score was called the diastolic function score and was also significantly different among the groups in the healthy population (Table 2). The prevalence of the participants who exceeded the upper limit partition value for each parameter was compared among the different age group, and the results were summarized in Fig 1 and Table 3.

**Table 2:** Changes in cardiac performance with aging (comparison of individual parameters between age groups). LVEF = left ventricular ejection fraction, A-L = area-length method, MV = mitral valve, E = early diastolic flow velocity, A = late diastolic flow velocity, e’ = mitral annulus early diastolic velocity, a’ = mitral annulus late diastolic velocity, DT = deceleration time

Parameters	Gr 1		Gr 2		Gr 3		Gr 4 + 5		Gr 4		Gr 5	
	mean	SD	mean	SD	mean	SD	mean	SD	mean	SD	mean	SD
LVEF (A-L) (%)	57.32	4.32	60.95*	5.78	62.59^†^	6.51	62.33	7.01	62.85	6.83	60.76	7.42
MV E/A ratio	1.85	0.35	1.51*	0.32	1.16**	0.34	0.76#	0.23	0.8	0.23	0.67##	0.2
MV e'/a' ratio (septal)	1.8	0.39	1.32*	0.4	0.88**	0.27	0.6#	0.18	0.62	0.19	0.55##	0.14
MV e'/a' ratio (lateral)	2.22	0.53	1.64*	0.51	1.17**	0.4	0.64#	0.25	0.68	0.26	0.52^‡^	0.17
MV E/e' ratio (septal)	6.35	1.03	7.71*	1.54	9.06**	2.26	13.72#	4.3	13.02	3.67	15.72##	5.3
MV E/e' ratio (lateral)	4.98	0.79	5.83*	1.11	6.85**	1.84	10.68#	3.71	10.26	3.41	11.89	4.29
MV DT (ms)	173.2	24.9	181.6	34.6	204.9**	36.1	236.4#	47.4	229.83	46.21	255.32##	46.53
Diastolic function score (mean value)	0.02	0.14	0.45*	0.95	3.06**	2.77	8.22#	2.37	7.81	2.5	9.39^‡^	1.43

*p < 0.01 vs. Gr 1;

**p < 0.01 vs. Gr 2;

^†^ *p* = 0.05 vs. Gr 2;

^#^ *p* < 0.01 vs. Gr 3;

^##^ *p* < 0.05 vs. Gr 4;

^‡^ *p* < 0.01 vs. Gr 4

**Table 3:** Comparison of individual indices of LV diastolic function between age groups in the healthy population. Abbreviations are the same as in Table 2

Parameters	G1 (%)	G2 (%)	G3 (%)	Gr 4 +5 (%)
MV E/A (<1)	0	3.67	31.86*	85**
e'/a': Sept (≤ 0.8)	0	6.42	47.79*	89.17**
e'/a': Lat (≤ 1.0)	0	6.42	33.63*	91.67**
E/e': Sept (10–15)	0	4.59	30.09*	53.33**
E/e': Sept (>15)	0	0	0.88	28.33**
E/e': Lat (8–15)	0	4.59	27.43*	63.33**
E/e': Lat (>15)	0	0	0	13.33**
MV DT (>240)	1.96	1.83	14.16*	43.33**
Score 0–2 number (%)	100	94.5	46.02*	2.5**
Score 3–5 number (%)	0	5.5	32.74*	15#
Score 6–10 number (%)	0	0	21.24*	82.5**

*p < 0.01 vs. Gr 2;

**p < 0.01 vs. Gr 3;

^#^ *p* < 0.05 vs. Gr 3

Taken together, the indices of LV diastolic function revealed significant differences between the younger groups (Gr2 vs. Gr3) and between the older group (Gr3 vs. Gr4 + Gr 5) in the criteria for E/A ratio < 1 (Fig 1A and Table 3), septal e’/a’ ratio ≤ 0.8 (Fig 1B), lateral e’/a’ ratio ≤ 1 (Fig 1B), septal E/e’ ratio: 10–15 (Fig 1C), lateral E/e’ ratio: 8–15 (Fig 1D), and deceleration time > 240 ms (Fig 1E). Significant differences in the indices of septal E/e’ ratio > 15 (Fig 1C) and lateral E/e’ ratio >15 (Fig 1D) (Gr 3 vs. Gr 4 + Gr 5: *P* < 0.01 for both, Table 3) were also found among the groups.

### Comparison and correlation between genders and diastolic function in healthy population

The comparison of all indices for the prevalence of the participants exceeding the upper limit partition value between the gender groups was presented in Table 4. In Gr 3, a septal E/e’ ratio of 10–15 and a lateral E/e’ ratio of 8–15 were more prevalent among females than males. Similar significant differences were also found for a septal E/e’ ratio >15 and a lateral E/e’ ratio >15 among Gr 4+5 (Table 4).

**Table 4:** Comparison of individual indices of LV diastolic function between genders in the healthy population. Abbreviations are the same as in Table 2

	Gr 1 Male (%)	Gr 1 Female (%)	Gr 2 Male (%)	Gr 2 Female (%)	Gr 3 Male (%)	Gr 3 Female (%)	Gr 4 + 5 Male (%)	Gr 4 + 5 Female (%)
MV E/A (<1)	0	0	0	5.41	26.1	35.82	80.9	87.67
e'/a': Sept (≤ 0.8)	0	0	14.3	2.7*	58.7	40.3	85.1	91.78
e'/a': Lat (≤ 1.0)	0	0	14.3	2.7*	28.3	37.31	89.4	93.15
E/e': Sept (10–15)	0	0	8.57	2.7	19.6	37.31**	51.1	54.79
E/e': Sept (>15)	0	0	0	0	0	1.49	12.8	38.36##
E/e': Lat (8–15)	0	0	2.86	5.41	13	37.31#	66	61.64
E/e': Lat (>15)	0	0	0	0	0	0	2.13	20.55##
MV DT (>240)	4.17	0	5.71	0*	28.3	4.48#	44.7	42.47
Score 0–2 number (%)	100	100	91.4	95.95	56.5	38.81	4.26	1.37
Score 3–5 number (%)	0	0	8.57	4.05	28.3	35.82	27.7	6.85##
Score 6–10 number (%)	0	0	0	0	15.2	25.37	68.1	91.78##

*p < 0.05 vs. Gr 2 Male;

**p < 0.05 vs. Gr 3 Male;

^#^ *p* < 0.05 vs. Gr 3 Male;

^##^ *p* < 0.05 vs. Gr 4 + Gr 5 Male

### Diastolic function score derived from the novel integrated score index

**Fig 2:**
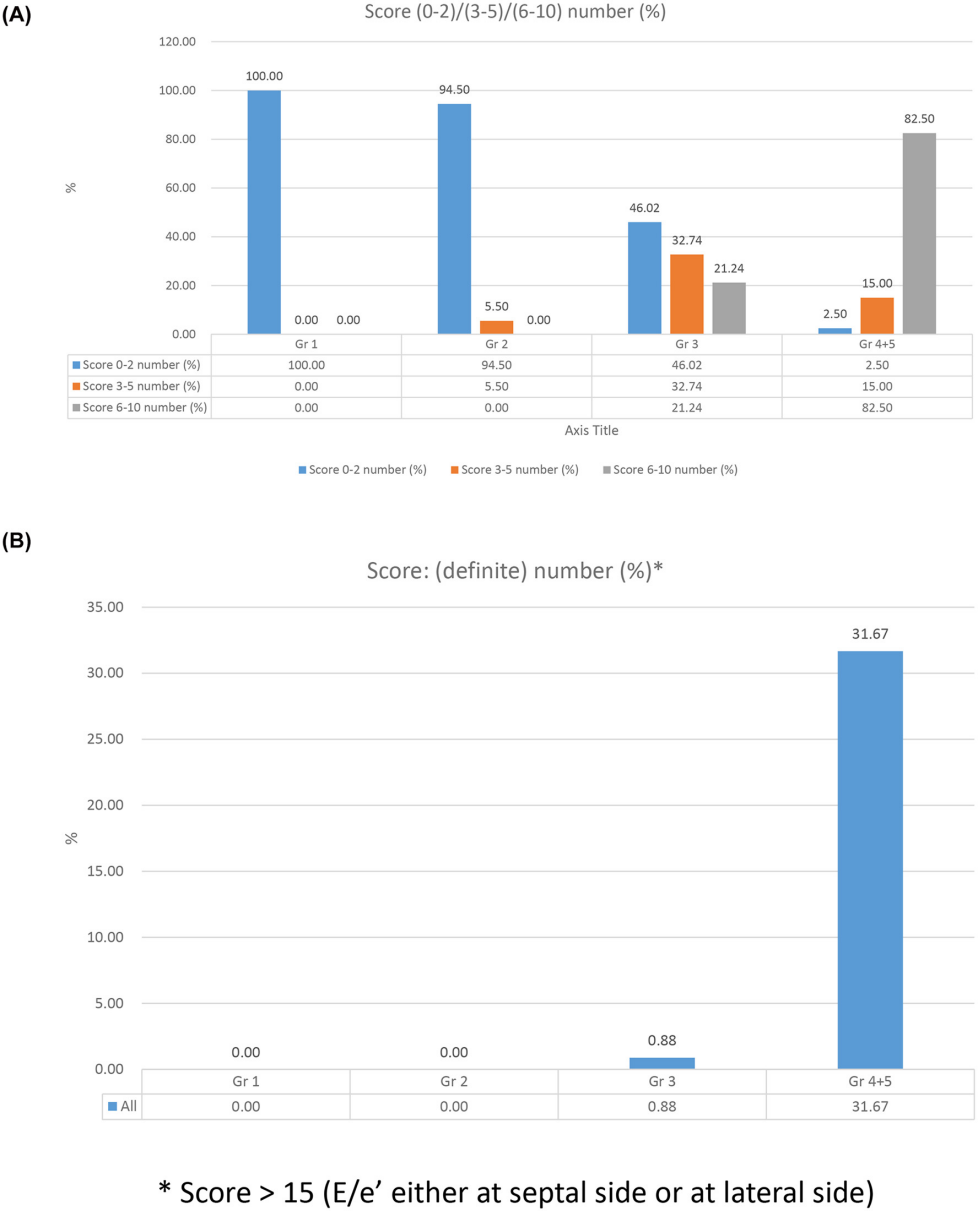
Left ventricular diastolic function, as assessed using the integrated score index: frequencies (%) of subjects with different scores in individual age groups within the healthy population. (A) scores of 0–2, 3–5, 6–10. (B) definitive score.

The frequencies of healthy subjects with different scores were presented in Table 3. Interestingly, we found that the diastolic function score was associated with the change in age. A score of 0–2 tended to be more prevalent in the group of younger subjects than in the group of elderly subjects (Gr 2 vs. Gr 3: *P* < 0.01; Gr 3 vs. Gr 4 + 5: P < 0.01) (Fig 2A and Table 3). A score of 3–5 was more prevalent in the transitional age group (Gr 3) (Gr 2 vs. Gr 3: *P*<0.01; Gr 3 vs. Gr 4+5: *P* = 0.02) (Fig 2A and Table 3). The high score categories (scores of 6–10) were significantly more frequent in the groups of elderly subjects (Gr 2 vs. Gr 3: *P* < 0.01; Gr 3 vs. Gr 4+5: *P* < 0.01) (Fig 2A and Table 3). Therefore, the difference in the score distribution was significantly associated with the change in age (Fig 2A). Especially, when the criteria of E/e’ > 15 (either at septal side or lateral side) was considered, it was shown to be highly prevalent in the elderly group (Gr 4+ Gr 5, age ≥ 70) (Fig 2B).

**Fig 3:**
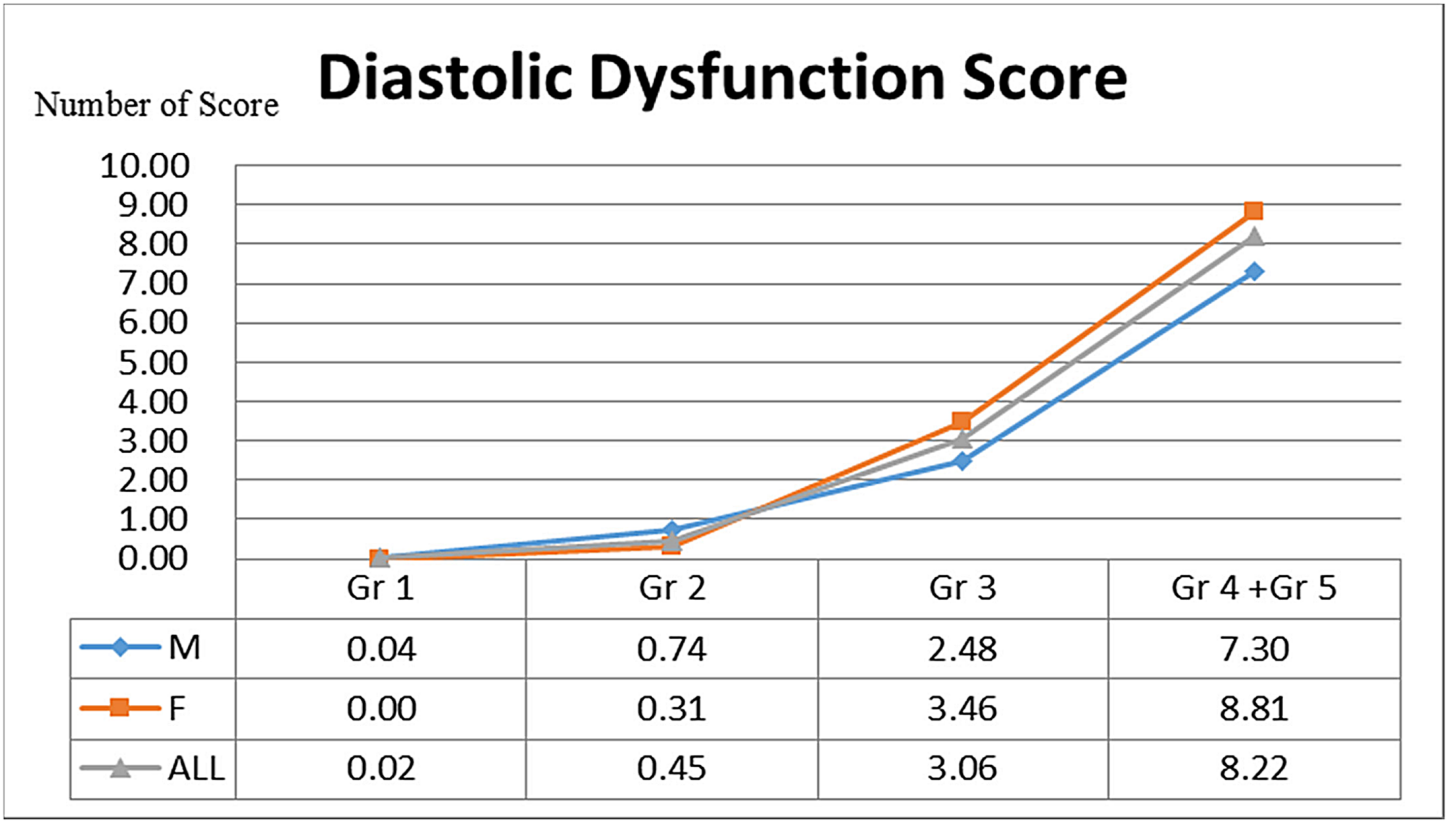
Integrated score index for the assessment of left ventricular diastolic function in the normal population (changes with age and gender).

We calculated the mean score and compared the values obtained for each age group in the healthy population. A higher mean score was associated with the groups of elderly subjects (Gr 2 vs. Gr 3: *P* = 0.00; Gr 3 vs. Gr 4+5: *P* = 0.00) (Fig 3 and Table 2). Comparisons of the frequencies of different scores between the genders are presented in Table 4. In the groups of elderly subjects (Gr 4 + 5), middle scores (scores of 3–5) were more prevalent among males (males vs. females: *P* < 0.01) (Table 4), whereas high scores (scores of 6–10) were more prevalent among females (for score 6–10, male vs. female: *P* < 0.01) (Table 4). The mean scores for each gender group among Gr 4 + 5 showed the same trend (male vs. female: *P* < 0.01) (Fig 3). These results suggested that diastolic dysfunction was more prevalent among elderly female subjects than younger women.

### Associations between conventional methodology suggested by the American Society of Echocardiography and the diastolic dysfunction score in discriminating diastolic dysfunction

**Fig 4:**
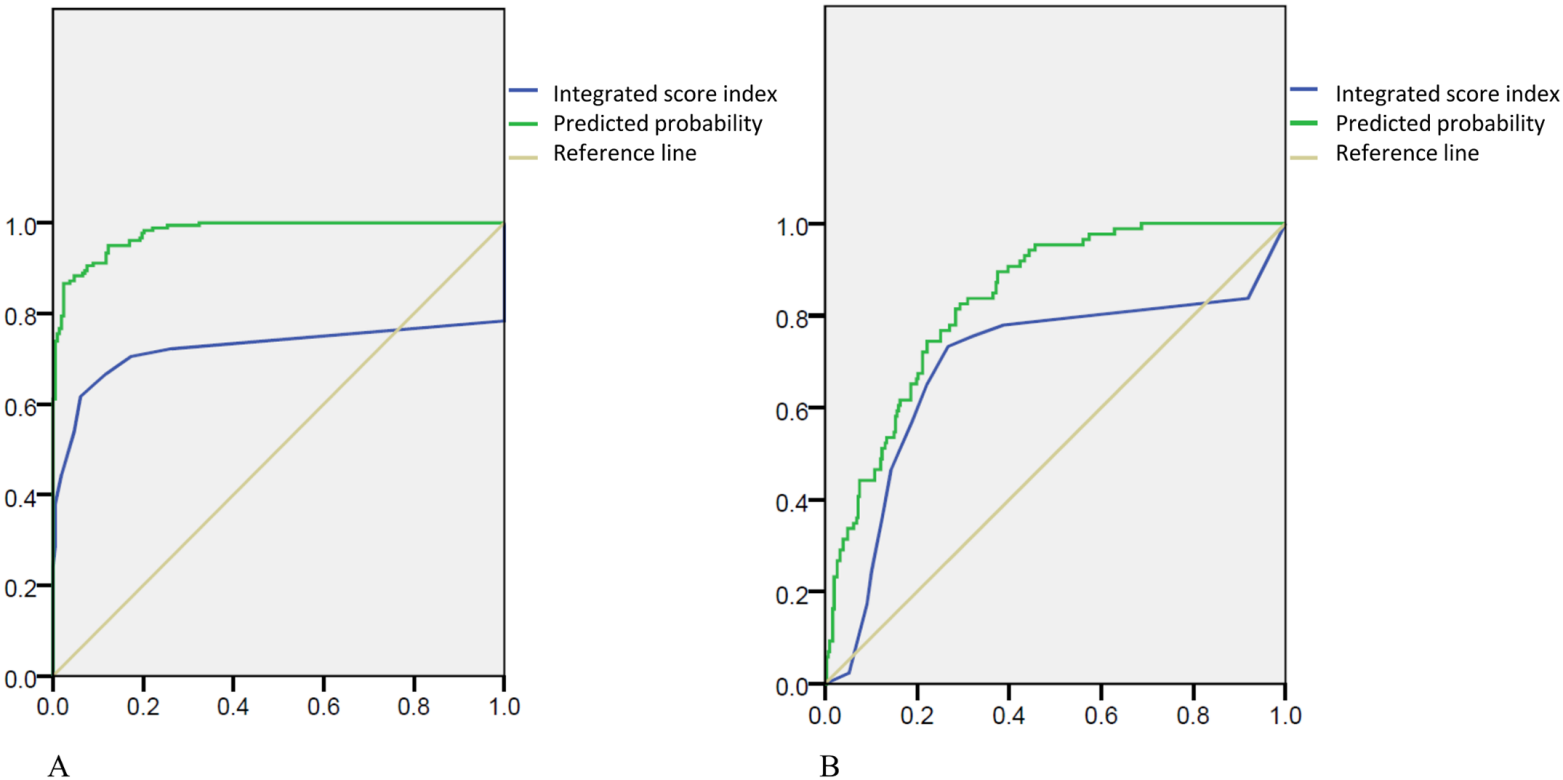
(A) Use of the integrated score index and predicted probability to discriminate any diastolic dysfunction (U.S. grades 1, 2, and 3) in the healthy population. Predicted value AUROC: 0.979; integrated score index AUROC: 0.722 (blue line: integrated score index; green line: predicted probability; yellow line: reference line). (B) Use of the integrated score index and predicted probability to discriminate moderate to severe diastolic dysfunction (U.S. grades 2 and 3) in the healthy population. Predicted value AUROC: 0.835; integrated score index AUROC: 0.692 (blue line: integrated score index; green line: predicted probability; yellow line: reference line).

Because the distribution of scores (scores of 0–2, scores of 3–5, scores of 6–10) was significantly different among the age groups in the healthy population, we defined four categories of diastolic function. A total score of 0–2 indicated normal diastolic function, a score of 3–5 was considered suggestive of LV diastolic dysfunction, and a score of 6–10 indicated impaired LV diastolic dysfunction in healthy subjects. The incidence of diastolic dysfunction, as assessed with the help of the diagnostic algorithm recommended by the American Society of Echocardiography [14] and determined using the diastolic function score derived using our integrated score counting method, was compared and analyzed using the ROC method. The AUROC of the integrated score index for predicting any diastolic dysfunction (Grade I, Grade II, and Grade III) discriminated by the grading of the American Society of Echocardiography [14] was 0.722, whereas the AUROC of the grading by the American Society of Echocardiography [14] was 0.979 (Fig 4). When moderate and severe diastolic dysfunction (Grade II and Grade III) were considered, the AUROC of the newly integrated score counting method was 0.692, and that of the grading by the American Society of Echocardiography [14] was 0.835 (Fig 4B).

### Odds ratio analysis for factors influencing the integrated score index

**Fig 5:**
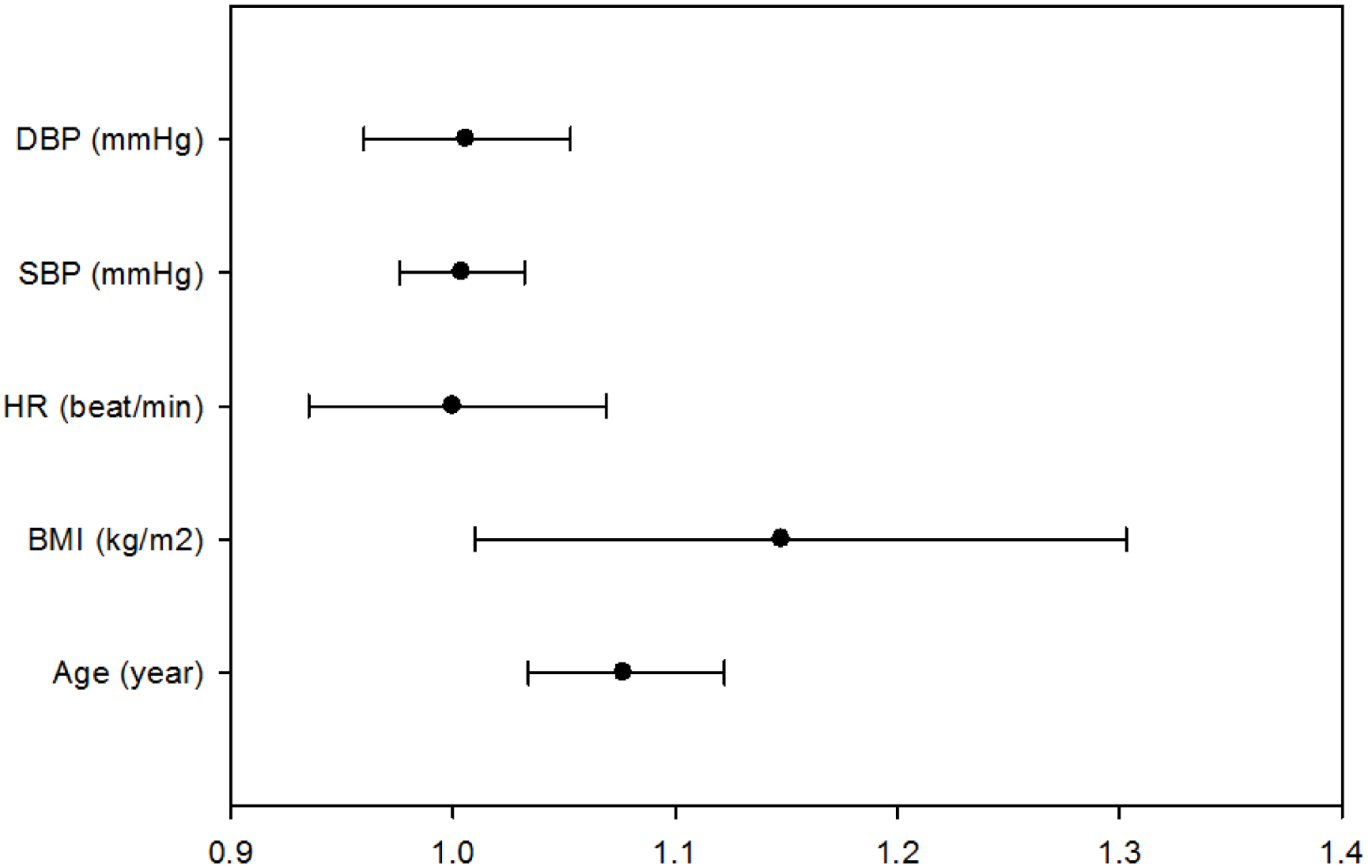
Odds ratio analysis for factors influencing the integrated scoring index. Age: 1.077 (CI: 1.034~1.122; *p* = 0.000); BMI: 1.148 (CI: 1.010~1.303; *p* = 0.034); HR: 1.000 (CI: 0.935~1.069; *p* = 0.998); SBP: 1.004 (CI: 0.976~ 1.032; *p* = 0.797); DBP: 1.006 (CI: 0.960~1.053; *p* = 0.807). BMI = body mass index, HR = heart rate, SBP = systolic blood pressure, DBP = diastolic blood pressure.

The influence of anthropometry on the score was associated with age (odd ratio: 1.077, CI: 1.034~1.122, *p* = 0.000) and BMI (odd ratio: 1.148, CI: 1.010~1.303, *p* = 0.034) after odds ratio analysis. (Fig 5)

### Clinical application of the integrated score index

The diastolic functions of individual subjects with hypertension (HTN), hypertrophic cardiomyopathy (HCM), and coronary artery disease (CAD) were evaluated by the integrated score index. The mean values of the diastolic function scores among the groups were 3.81 ± 0.12 in healthy subjects and 6.48 ± 0.19 in HTN, 7.35 ± 0.46 in HCM, and 6.62 ± 0.29 in CAD patients (Table 5). We then compared the diastolic function score of each group after adjusting for age, sex and BMI. The healthy subjects had lower diastolic function score compared with HTN (p = 0.00), HCM (p = 0.00), and CAD (p = 0.00) patients (Table 6). Thus, the integrated score index has good clinical applicability for discriminating patients with diseases with impaired diastolic function from healthy subjects.

**Table 5:** The mean values of the diastolic function scores among different groups. HTN = hypertension, HCM = hypertrophic cardiomyopathy, CAD = coronary artery disease

	Case (number)	Diastolic Function Score (mean)	SD	95% CI
Healthy	393	3.81	0.12	3.58~ 4.03
HTN	145	6.48	0.19	6.11~ 6.84
HCM	24	7.35	0.46	6.44~ 8.26
CAD	67	6.62	0.29	6.04~ 7.19

**Table 6:** Comparison of the diastolic function score within each group, A compared with B. A-B: A diastolic function score—B diastolic function score. Abbreviations are the same as in Table 5

A	B	A-B	SD	p- value	95% CI
Healthy	HTN	-2.67	0.22	0.00	-3.10 ~ -2.24
Healthy	HCM	-3.54	0.48	0.00	-4.49~ -2.60
Healthy	CAD	-2.81	0.32	0.00	-3.44~ -2.18
HTN	HCM	-0.87	0.50	0.08	-1.85~ 0.11
HTN	CAD	-0.14	0.35	0.69	-0.82~ 0.54
HCM	CAD	0.73	0.54	0.17	-0.32~ 1.79

### Comparison of all subjects (healthy, HTN, HCM, CAD) with the integrated score index and the conventional methodology suggested by the American Society of Echocardiography for diastolic function

**Fig 6:**
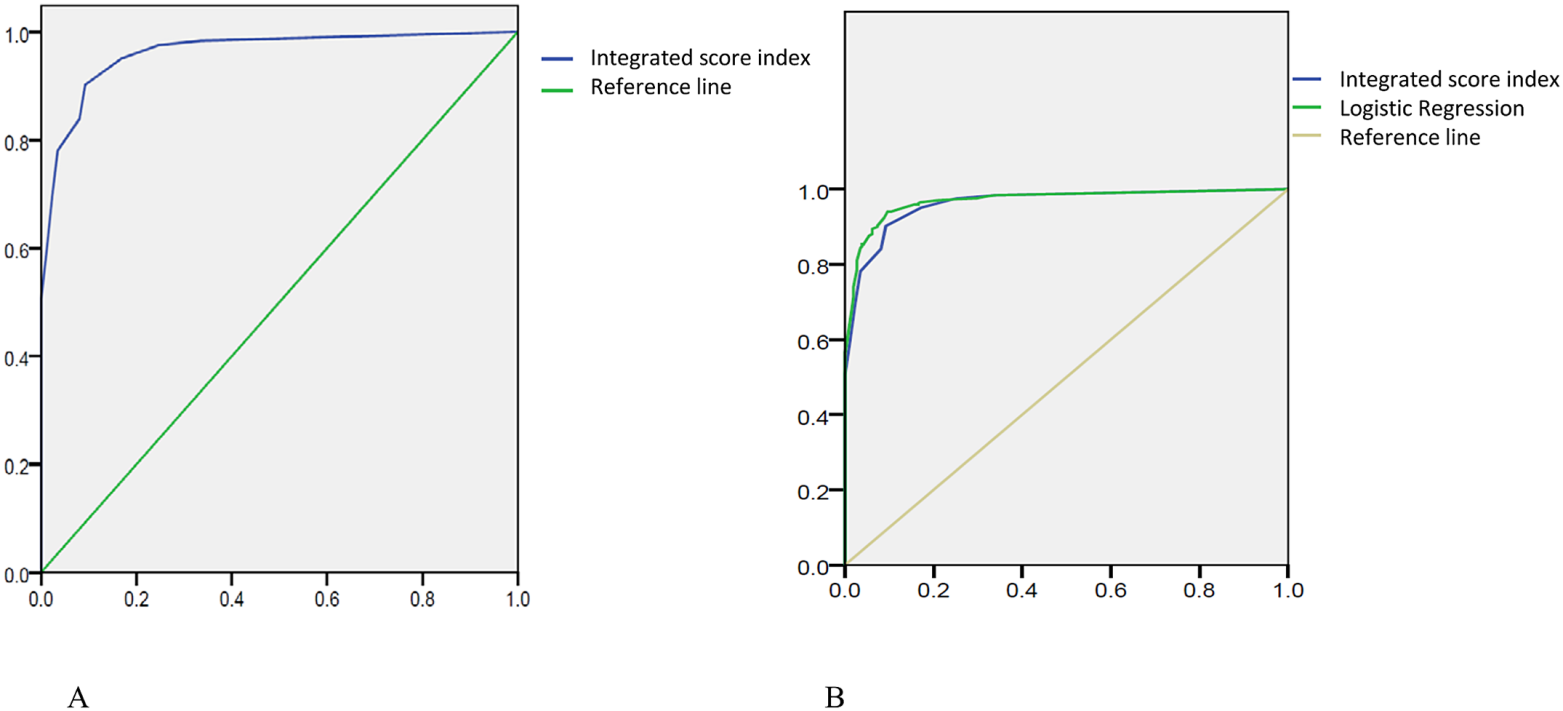
(A) Use of the integrated score index and predicted probability to discriminate any DHF in all the subjects (U.S. grades 1, 2, and 3). Integrated score index AUROC: 0.962 (blue line: integrated score index; green line: reference line). (B) The AUROC of the integrated score index used to predict any diastolic dysfunction (U.S. grades 1, 2, and 3) was 0.962, and the AUROC of the method suggested by the logistic regression was 0.970 (blue line: integrated score index; green line: logistic regression; yellow line: reference line).

We used all the subjects, including the healthy, HTN, HCM and CAD groups, for further analysis. Compared with that of the diagnostic algorithm recommended by the American Society of Echocardiography [14], the AUROC of our integrated score index used to predict any diastolic dysfunction (Grade I, Grade II, and Grade III) was 0.962 (Fig 6A).

By logistic regression analysis, we obtained the suggested scores of each parameter comprising the score index to evaluate the diastolic function. We then used those scores to evaluate the diastolic function of all the subjects individually and compared the results with the diagnostic algorithm recommended by the American Society of Echocardiography [14]. The AUROC of our integrated score index used to predict any diastolic dysfunction (Grade I, Grade II, and Grade III) was 0.962, and the AUROC was 0.970, based on logistic regression analysis (Fig 6B).

**Fig 7:**
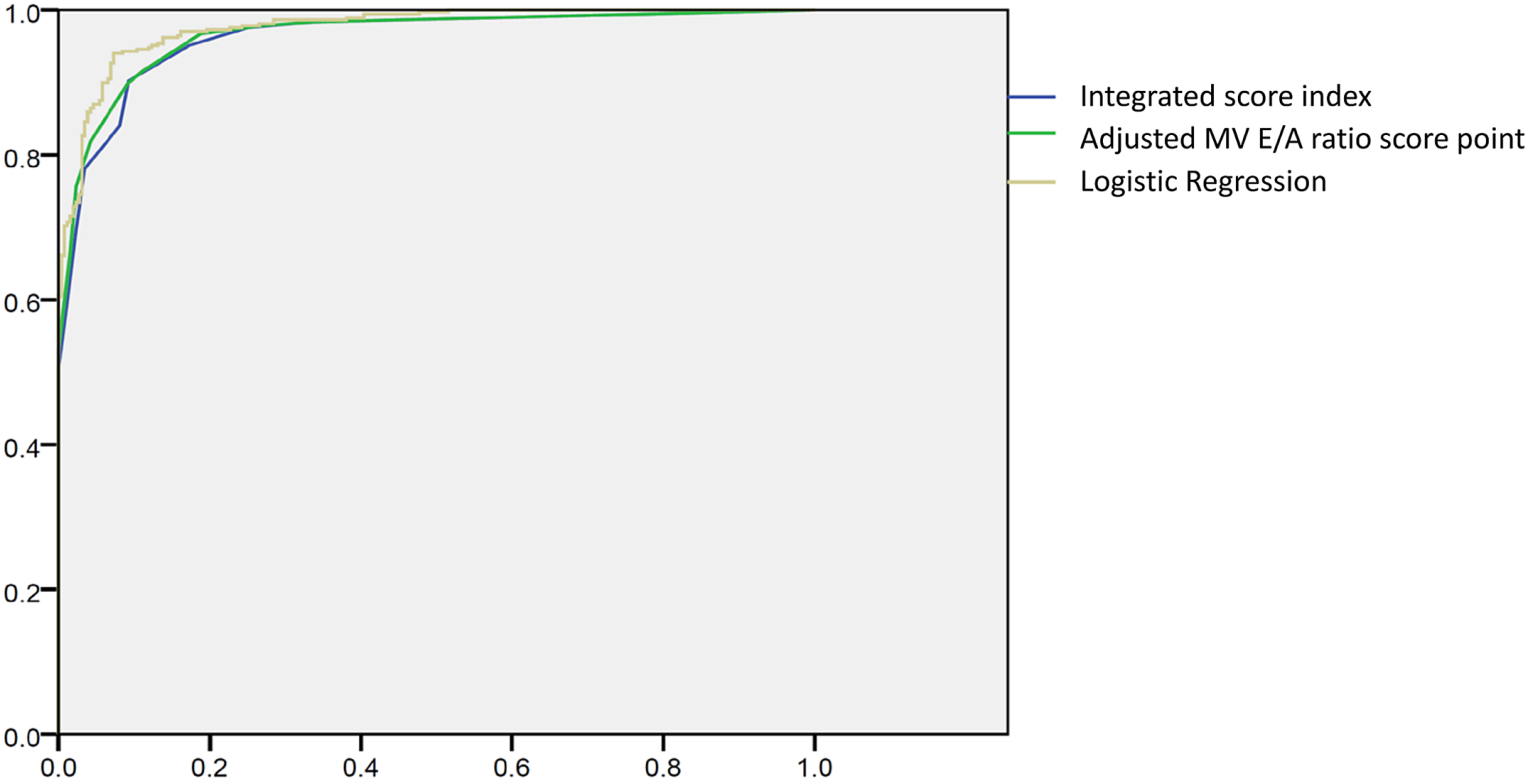
The AUROC of the integrated score index used to predict any diastolic dysfunction (U.S. grades 1, 2, and 3) was 0.962, the AUROC with the criteria of MV E/A Doppler <1 with a score of 2 was 0.965, and the AUROC of the method suggested by the logistic regression was 0.970 (blue line: integrated score index; green: adjusted MV E/A score point; yellow line: logistic regression).

Although pulsed wave (PW) Doppler is a reliable parameter for evaluating diastolic function, debate remains about the superiority of PW Doppler or tissue Doppler in evaluating diastolic function. We adjusted the grading of an MV E/A ratio <1 from a score of 1 to a score of 2, but the scores for other parameters were kept the same. The AUROC with the criteria of an MV E/A ratio <1 with a score of 2 was 0.965 (Fig 7; green line). Because our original integrated score index comprised several parameters from PW and tissue Doppler, we also gave the parameter “E/e’ ratio (either lateral or septal)” a higher score and integrated this score from both PW and tissue Doppler. This integration might explain why the results did not change dramatically after adjusting the score for grading the MV E/A ratio.

## Discussion

Studies in clinical cardiology have shown that myocardial function changes with age, even in healthy individuals [16], and that the prevalence of diastolic dysfunction also increases with age.[1] Kitzman et al. described the normal age-related changes in the anatomy of the heart that may affect diastolic function. [17] These alterations include changes in the myocardial composition (fibrosis and collagen accumulation, myocyte degeneration, and senile amyloid accumulation), cardiac structure (increasing cardiac mass, ventricular wall thickness, and AO dimension), valves, the pericardium, and vasculature.

Previous studies have concluded that diastolic dysfunction could be detected using invasive methods (LV end-diastolic pressure >16 mmHg or mean pulmonary capillary wedge pressure >12 mmHg).[5] Clinically, it is important to determine LV diastolic function noninvasively and accurately. Echocardiographic parameters, including those derived from MV flow velocity measurements such as E/A and DT, have been widely used for the comprehensive assessment of LV relaxation, LV diastolic stiffness, and LV filling pressures.[5] Analysis of the mitral inflow velocity provides useful information for the determination of filling pressures and for predicting the prognosis in selected patients. [18] However, there were several interrelated factors on mitral inflow such as the ventricular relaxation rate, diastolic suction, and atrial compliance.[18] Studies have found that analysis of the mitral flow wave alone, with 40% to 70% specificity, could not reliably be used to diagnose diastolic dysfunction.[19] Sohn et al.[20] also described a “pseudonormalization” of diastolic function by PW Doppler of mitral inflow. [21] Therefore, Kasner et al.[19] did not recommend the single use of PW-Doppler mitral inflow measurements and proposed the LV filling index E/e’ lateral as the best index to detect diastolic dysfunction in patients with heart failure and a normal ejection fraction; Ommen et al. [18] also made a similar suggestion.[21] Other studies also showed that indices derived from TDI were excellent predictors of the diastolic filling pressure [12] and were key elements in the diagnosis and management of diastolic dysfunction.[10, 18]

To overcome the limitations of the individual parameters derived from echocardiography and to improve the complex diagnostic algorithm recommended by the current guidelines for the echocardiographic assessments of diastolic function, [14, 15] we proposed a new diagnostic modality for evaluating and grading the degree of diastolic function.

In our studies, we established the average value of each echocardiographic parameter for evaluating cardiac structure and function in a group of subjects with a wide age range, including subjects over 90 years old. Our results showed that advanced age was associated with higher SBP, higher AO root dimension, and increased posterior wall thickness. The pressure gradients of MV, TV, and AV were also higher in the group of elderly subjects. However, no significant difference was found between LA and LV size of the young and elderly subjects.

We also established the age dependence of Doppler diastolic function parameters in a Taiwanese population. The indices corresponding to an E/A ratio <1, a deceleration time >240 ms, a septal e’/a’ ratio ≤ 0.8, a lateral e’/a’ ratio ≤ 1, a septal E/e’ ratio 10–15, a lateral E/e’ ratio 8–15, a septal E/e’ ratio >15, and a lateral E/e’ ratio >15 in the elderly subjects were significantly different from those of the younger subjects. In this study, gender-specific differences in the indices of LV diastolic function within the age groups were compared. Among female subjects, there was a significantly greater prevalence rate of a septal E/e’ ratio of 10–15 or a lateral E/e’ ratio of 8–15 among the middle age group and a septal or lateral E/e’ ratio >15 among the elderly subject group.

Our integrated score index comprised indices derived based on the effects of aging, as mentioned above. The mitral flow velocity and tissue Doppler parameters were incorporated in our integrated score index. This integrated score index was also used to evaluate the healthy participants in this study. Interesting, we found that the echocardiographic measurement corresponding to an E/e’ > 15 (either at septal side or lateral side) was significantly useful for the elderly subjects (Gr 4+5, age ≥ 70 years old) (Fig 2B) in this study. In previous studies, if the ratio of early mitral inflow to early mitral annulus velocity (E/e’ ratio) was 8–15, additional non-invasive methods were required to document diastolic dysfunction.[5] However, if the E/e’ ratio was >15, it was suggested as a useful index to assess diastolic dysfunction.[22, 23] This measure was also endorsed by the European and American consensus statements on diastolic dysfunction; [24] thus, we defined this indices as the diagnosis of definite diastolic dysfunction and gave a score of 10 for this grading regardless of other measurements of the score index.

The score distribution associated with advancing age was significantly different. High scores or “definite groups” were more frequent in the group of elderly subjects. The results derived from the integrated score index were consistent with the changes in diastolic function in the different age groups. Within the group of elderly subjects, high scores or “definite groups” were more frequent among female subjects. Our results suggested that within the group of elderly subjects, diastolic dysfunction was more prevalent among the female gender group.

We also applied this integrated score index for clinical application in subjects likely to have impaired diastolic function. Our score index showed good discriminatory ability to distinguish healthy subjects from those with diastolic dysfunction such as HTN, HCM, and CAD patients. When the total sum of scores was equal to or greater than 4, the score index could discriminate subjects with diseases with impaired diastolic function from the healthy subjects.

The results derived by using our method showed good correlation with those obtained using the diagnostic algorithm recommended in the current American Society of Echocardiography guidelines for documenting diastolic dysfunction. [14] This integrated score index also showed good correlation with the method suggested by the logistic regression analysis for evaluating diastolic function. Therefore, this integrated score index indicates normal diastolic function with a total score equal to or less than 2 and impaired LV diastolic dysfunction with a score equal to or greater than 6 in healthy subjects. If the total sum of the score index is equal to or greater than 4, this score index could discriminate the healthy population from subjects who possibly have diastolic dysfunction such as HTN, HCM, and CAD patients. This integrated score index is easier to memorize and is more user friendly, providing the possibility of quantifying diastolic function by the obtained score. This is particularly helpful for predicting the risk of diastolic dysfunction and for clinical application.

## Conclusions

The early identification of diastolic dysfunction in asymptomatic patients may provide an opportunity to appropriately manage the underlying etiology and to prevent progression to advanced HF. If already known analysis methods cannot lead to a clear diagnosis of diastolic dysfunction, our integrated score index might provide an another avenue for the comprehensive evaluation of diastolic function. Our index might also be used carefully to stratify cardiac function during aging, which would facilitate the performance of further studies of diastolic function and would make discriminating diseases with impaired diastolic function easier.

## Supporting Information

S1 File Changes in cardiac structures with aging in both genders and in all healthy subjects. (DOCX)

S2 File Basic characteristics of the subjects with HTN, HCM, CAD. (DOCX)

S3 File Medications for all the subjects. (DOCX)
