# Supplementary material for: A Novel Integrated Score Index of Echocardiographic Indices for the Evaluation of Left Ventricular Diastolic Function
Source: PLoS One. 2015 Nov 10;10(11):e0142175. doi: 10.1371/journal.pone.0142175 (PMC4640516; doi:10.1371/journal.pone.0142175)
Supplement: S1 File — (DOCX) [file pone.0142175.s001.docx]

**Supplemental Material 1:** Changes in cardiac structures with aging in both genders and in all healthy subjects

|  | Gr 1 (age ≤29 yr) | | | | | | | | | Gr 2 (age 30–49 yr) | | | | | | | | |
| --- | --- | --- | --- | --- | --- | --- | --- | --- | --- | --- | --- | --- | --- | --- | --- | --- | --- | --- |
|  | Male | | | Female | | | All | | | Male | | | Female | | | All | | |
| Parameter | N | mean | SD | N | mean | SD | N | mean | SD | N | mean | SD | N | mean | SD | N | mean | SD |
| Age (yr) | 24 | 23.88 | 2.25 | 27 | 25.67 | 2.95 | 51 | 24.82 | 2.77 | 35 | 40.20 | 6.02 | 74 | 40.08 | 5.87 | 109 | 40.12 | 5.89 |
| SBP (mmHg) | 23 | 123.48 | 8.27 | 27 | 110.78 | 9.89 | 50 | 116.62 | 11.11 | 35 | 125.71 | 9.88 | 74 | 117.58 | 11.39 | 109 | 120.19 | 11.53 |
| DBP (mmHg) | 23 | 76.17 | 5.19 | 27 | 71.30 | 10.61 | 50 | 73.54 | 8.83 | 35 | 82.23 | 6.10 | 74 | 76.30 | 7.77 | 109 | 78.20 | 7.76 |
| BMI (kg/m^2^) | 24 | 22.06 | 2.73 | 27 | 20.62 | 2.42 | 51 | 21.30 | 2.65 | 35 | 24.83 | 2.39 | 74 | 21.18 | 2.61 | 109 | 22.35 | 3.06 |
| HR (beats/min) | 24 | 68 | 7.88 | 27 | 69 | 8.34 | 51 | 68 | 8.06 | 35 | 69 | 5.78 | 74 | 68 | 5.27 | 109 | 68 | 5.47 |
| LVEDD (cm) | 24 | 4.98 | 0.31 | 27 | 4.47 | 0.39 | 51 | 4.71 | 0.44 | 35 | 4.74 | 0.34 | 74 | 4.41 | 0.32 | 109 | 4.52 | 0.36 |
| LVESD (cm) | 24 | 3.00 | 0.27 | 27 | 2.66 | 0.37 | 51 | 2.82 | 0.37 | 35 | 2.73 | 0.38 | 74 | 2.52 | 0.34 | 109 | 2.59 | 0.37 |
| LA (cm) | 24 | 3.05 | 0.34 | 27 | 2.85 | 0.32 | 51 | 2.95 | 0.34 | 35 | 3.34 | 0.39 | 74 | 2.93 | 0.39 | 109 | 3.06 | 0.43 |
| AO root (cm) | 24 | 2.88 | 0.33 | 27 | 2.53 | 0.26 | 51 | 2.70 | 0.34 | 35 | 3.07 | 0.32 | 74 | 2.68 | 0.35 | 109 | 2.81 | 0.39 |
| IVS (cm) | 24 | 0.78 | 0.08 | 27 | 0.72 | 0.07 | 51 | 0.75 | 0.08 | 35 | 0.90 | 0.13 | 74 | 0.74 | 0.10 | 109 | 0.79 | 0.13 |
| PW (cm) | 24 | 0.76 | 0.08 | 27 | 0.69 | 0.07 | 51 | 0.72 | 0.08 | 35 | 0.84 | 0.12 | 74 | 0.71 | 0.07 | 109 | 0.76 | 0.11 |
| MVPG (mmHg) | 23 | 2.43 | 0.54 | 21 | 3.11 | 0.95 | 44 | 2.75 | 0.83 | 21 | 2.58 | 0.78 | 59 | 2.68 | 0.76 | 80 | 2.65 | 0.76 |
| TV PG (mmHg) | 23 | 18.37 | 3.23 | 25 | 16.70 | 2.57 | 48 | 17.50 | 2.99 | 30 | 18.17 | 3.48 | 68 | 18.13 | 3.43 | 98 | 18.14 | 3.43 |
| AV PG (mmHg) | 24 | 5.39 | 2.02 | 23 | 5.53 | 1.06 | 47 | 5.46 | 1.61 | 26 | 5.01 | 1.17 | 70 | 5.63 | 1.43 | 96 | 5.46 | 1.39 |

yr = years, SBP = systolic blood pressure, DBP = diastolic blood pressure, BMI = body mass index, HR= heart rate, LVEDD = left ventricular end-diastolic dimension, LVESD = left ventricular end-systolic dimension, LA = left atrium, AO = aortic root dimension, IVS = interventricular septum, PW = posterior wall, MV = mitral valve, TV = tricuspid valve, AV = aortic valve, PG = pressure gradient, Gr = group, N = number, SD = standard deviation

**Supplemental Material 1:** Changes in cardiac structures with aging in both genders and in all healthy subjects

|  | Gr 3 (age 50–69 yr) | | | | | | | | | Gr 4 (age 70–89 yr) | | | | | | | | |
| --- | --- | --- | --- | --- | --- | --- | --- | --- | --- | --- | --- | --- | --- | --- | --- | --- | --- | --- |
|  | Male | | | Female | | | All | | | Male | | | Female | | | All | | |
| Parameter | N | mean | SD | N | mean | SD | N | mean | SD | N | mean | SD | N | mean | SD | N | mean | SD |
| Age (yr) | 46 | 57.63 | 5.33 | 67 | 58.61 | 4.73 | 113 | 58.21 | 4.98 | 34 | 80.56 | 5.44 | 55 | 80.44 | 5.50 | 89 | 80.48 | 5.45 |
| SBP (mmHg) | 45 | 125.04 | 12.94 | 67 | 126.34 | 14.91 | 112 | 125.82 | 14.10 | 33 | 143.88 | 18.07 | 55 | 145.73 | 18.24 | 88 | 145.03 | 18.09 |
| DBP (mmHg) | 45 | 82.29 | 8.38 | 67 | 78.12 | 7.85 | 112 | 79.79 | 8.29 | 33 | 74.88 | 8.82 | 55 | 76.02 | 8.30 | 88 | 75.59 | 8.47 |
| BMI (kg/m^2^) | 46 | 23.49 | 2.31 | 67 | 23.11 | 2.43 | 113 | 23.26 | 2.38 | 34 | 23.40 | 3.16 | 55 | 23.97 | 3.36 | 89 | 23.75 | 3.28 |
| HR (beats/min) | 46 | 68 | 5.20 | 67 | 67 | 4.95 | 113 | 68 | 5.10 | 34 | 67 | 5.86 | 55 | 67 | 5.81 | 89 | 67 | 5.49 |
| LVEDD (cm) | 46 | 4.67 | 0.37 | 67 | 4.43 | 0.34 | 113 | 4.53 | 0.37 | 34 | 4.84 | 0.50 | 55 | 4.44 | 0.44 | 89 | 4.59 | 0.50 |
| LVESD (cm) | 46 | 2.55 | 0.34 | 67 | 2.30 | 0.37 | 113 | 2.40 | 0.38 | 34 | 2.60 | 0.48 | 55 | 2.23 | 0.42 | 89 | 2.37 | 0.48 |
| LA (cm) | 46 | 3.27 | 0.37 | 67 | 3.27 | 0.38 | 113 | 3.27 | 0.38 | 34 | 3.43 | 0.57 | 55 | 3.39 | 0.37 | 89 | 3.41 | 0.45 |
| AO root (cm) | 46 | 3.47 | 0.35 | 67 | 2.96 | 0.35 | 113 | 3.17 | 0.43 | 34 | 3.51 | 0.32 | 55 | 3.17 | 0.42 | 89 | 3.30 | 0.42 |
| IVS (cm) | 46 | 0.94 | 0.13 | 67 | 0.83 | 0.11 | 113 | 0.88 | 0.13 | 34 | 0.99 | 0.24 | 55 | 0.92 | 0.20 | 89 | 0.95 | 0.21 |
| PW (cm) | 46 | 0.87 | 0.11 | 67 | 0.79 | 0.11 | 113 | 0.82 | 0.11 | 34 | 0.89 | 0.14 | 55 | 0.85 | 0.15 | 89 | 0.87 | 0.15 |
| MVPG (mmHg) | 14 | 2.37 | 1.09 | 39 | 2.80 | 0.75 | 53 | 2.68 | 0.86 | 25 | 3.44 | 0.73 | 38 | 4.52 | 1.80 | 63 | 4.09 | 1.55 |
| TV PG (mmHg) | 38 | 19.84 | 3.71 | 66 | 20.47 | 3.72 | 104 | 20.24 | 3.71 | 34 | 24.00 | 4.17 | 55 | 24.79 | 5.32 | 89 | 24.49 | 4.91 |
| AV PG (mmHg) | 29 | 4.87 | 1.92 | 56 | 6.47 | 1.72 | 85 | 5.93 | 1.94 | 34 | 8.14 | 3.21 | 54 | 9.62 | 3.79 | 88 | 9.05 | 3.64 |

yr = years, SBP = systolic blood pressure, DBP = diastolic blood pressure, BMI = body mass index, HR= heart rate, LVEDD = left ventricular end-diastolic dimension, LVESD = left ventricular end-systolic dimension, LA = left atrium, AO = aortic root dimension, IVS = interventricular septum, PW = posterior wall, MV = mitral valve, TV = tricuspid valve, AV = aortic valve, PG = pressure gradient, Gr = group, N = number, SD = standard deviation

**Supplemental Material 1:** Changes in cardiac structures with aging in both genders and in all healthy subjects

|  | Gr 5 (age ≥90 yr) | | | | | | | | | Gr 4 + 5 (age ≥70 yr) | | | | | | | | |
| --- | --- | --- | --- | --- | --- | --- | --- | --- | --- | --- | --- | --- | --- | --- | --- | --- | --- | --- |
|  | Male | | | Female | | | All | | | Male | | | Female | | | All | | |
| Parameter | N | mean | SD | N | mean | SD | N | mean | SD | N | mean | SD | N | mean | SD | N | mean | SD |
| Age (yr) | 13 | 93.38 | 2.79 | 18 | 93.83 | 4.00 | 31 | 93.65 | 3.50 | 47 | 84.11 | 7.54 | 73 | 83.74 | 7.77 | 120 | 83.88 | 7.65 |
| SBP (mmHg) | 13 | 143.92 | 18.04 | 18 | 144.89 | 19.46 | 31 | 144.48 | 18.58 | 46 | 143.89 | 17.86 | 73 | 145.52 | 18.41 | 119 | 144.89 | 18.14 |
| DBP (mmHg) | 13 | 76.00 | 10.37 | 18 | 73.11 | 9.21 | 31 | 74.32 | 9.65 | 46 | 75.20 | 9.18 | 73 | 75.30 | 8.56 | 119 | 75.26 | 8.77 |
| BMI (kg/m^2^) | 13 | 22.47 | 2.90 | 18 | 24.27 | 2.49 | 31 | 23.52 | 2.78 | 47 | 23.14 | 3.09 | 73 | 24.05 | 3.15 | 120 | 23.69 | 3.15 |
| HR (beats/min) | 13 | 67 | 3.53 | 18 | 67 | 5.48 | 31 | 67 | 4.63 | 47 | 67 | 5.52 | 73 | 67 | 5.14 | 120 | 67 | 5.32 |
| LVEDD (cm) | 13 | 4.50 | 0.37 | 18 | 4.34 | 0.51 | 31 | 4.41 | 0.46 | 47 | 4.74 | 0.49 | 73 | 4.42 | 0.45 | 120 | 4.54 | 0.49 |
| LVESD (cm) | 13 | 2.54 | 0.39 | 18 | 2.36 | 0.38 | 31 | 2.43 | 0.38 | 47 | 2.59 | 0.45 | 73 | 2.26 | 0.41 | 120 | 2.39 | 0.45 |
| LA (cm) | 13 | 3.20 | 0.74 | 18 | 3.61 | 0.71 | 31 | 3.44 | 0.74 | 47 | 3.37 | 0.62 | 73 | 3.45 | 0.48 | 120 | 3.41 | 0.54 |
| AO root (cm) | 13 | 3.83 | 0.49 | 18 | 3.33 | 0.50 | 31 | 3.54 | 0.54 | 47 | 3.60 | 0.39 | 73 | 3.21 | 0.44 | 120 | 3.36 | 0.46 |
| IVS (cm) | 13 | 1.11 | 0.25 | 18 | 1.05 | 0.25 | 31 | 1.08 | 0.25 | 47 | 1.02 | 0.24 | 73 | 0.95 | 0.22 | 120 | 0.98 | 0.23 |
| PW (cm) | 13 | 0.95 | 0.12 | 18 | 0.92 | 0.13 | 31 | 0.93 | 0.13 | 47 | 0.91 | 0.14 | 73 | 0.87 | 0.15 | 120 | 0.88 | 0.15 |
| MVPG (mmHg) | 12 | 3.90 | 1.41 | 14 | 5.28 | 1.91 | 26 | 4.64 | 1.81 | 37 | 3.59 | 1.01 | 52 | 4.73 | 1.84 | 89 | 4.25 | 1.64 |
| TV PG (mmHg) | 13 | 25.23 | 6.11 | 18 | 27.13 | 7.15 | 31 | 26.33 | 6.69 | 47 | 24.34 | 4.75 | 73 | 25.37 | 5.86 | 120 | 24.97 | 5.45 |
| AV PG (mmHg) | 12 | 8.24 | 4.73 | 18 | 15.86 | 9.05 | 30 | 12.81 | 8.42 | 46 | 8.17 | 3.61 | 72 | 11.18 | 6.14 | 118 | 10.01 | 5.49 |

yr = years, SBP = systolic blood pressure, DBP = diastolic blood pressure, BMI = body mass index, HR= heart rate, LVEDD = left ventricular end-diastolic dimension, LVESD = left ventricular end-systolic dimension, LA = left atrium, AO = aortic root dimension, IVS = interventricular septum, PW = posterior wall, MV = mitral valve, TV = tricuspid valve, AV = aortic valve, PG = pressure gradient, Gr = group, N = number, SD = standard deviation
