# Supplementary material for: A Novel Integrated Score Index of Echocardiographic Indices for the Evaluation of Left Ventricular Diastolic Function
Source: PLoS One. 2015 Nov 10;10(11):e0142175. doi: 10.1371/journal.pone.0142175 (PMC4640516; doi:10.1371/journal.pone.0142175)
Supplement: S2 File — (DOCX) [file pone.0142175.s002.docx]

**Supplemental Material 2:** Basic characteristics of the subjects with HTN, HCM, CAD

|  | HTN | | | | | | | | | HCM | | | | | | | | |
| --- | --- | --- | --- | --- | --- | --- | --- | --- | --- | --- | --- | --- | --- | --- | --- | --- | --- | --- |
|  | Male | | | Female | | | All | | | Male | | | Female | | | All | | |
| Parameter | N | mean | SD | N | mean | SD | N | mean | SD | N | mean | SD | N | mean | SD | N | mean | SD |
| Age (yr) | 76 | 58.80 | 10.80 | 69 | 61.97 | 7.37 | 145 | 60 | 9.4 | 19 | 61.63 | 9.23 | 5 | 65.8 | 3.27 | 24 | 63 | 8.5 |
| SBP (mmHg) | 76 | 147.38 | 13.72 | 69 | 153.32 | 18.39 | 145 | 150 | 17 | 19 | 133.63 | 14.88 | 5 | 150.4 | 23.12 | 24 | 137 | 18 |
| DBP (mmHg) | 76 | 91.68 | 10.53 | 69 | 90.70 | 10.54 | 145 | 91 | 11 | 19 | 81.21 | 6.94 | 5 | 82.2 | 8.81 | 24 | 81 | 7.2 |
| BMI (kg/m^2^) | 76 | 25.66 | 2.30 | 69 | 24.14 | 2.64 | 145 | 24.9 | 2.57 | 19 | 25.37 | 2.59 | 5 | 26.44 | 1.70 | 24 | 25.6 | 2.44 |
| HR (beats/min) | 76 | 67 | 5.95 | 69 | 73 | 7.49 | 145 | 70 | 7 | 19 | 70 | 9.66 | 5 | 68 | 11.07 | 24 | 69 | 10.32 |

HTN = hypertension, HCM = hypertrophic cardiomyopathy, CAD = coronary artery disease, yr = years, SBP = systolic blood pressure, DBP = diastolic blood pressure, BMI = body mass index, HR= heart rate, Gr = group, N = number, SD = standard deviation

**Supplemental Material 2:** Basic characteristics of the subjects with HTN, HCM, CAD

|  | CAD | | | | | | | | |
| --- | --- | --- | --- | --- | --- | --- | --- | --- | --- |
|  | Male | | | Female | | | All | | |
| Parameter | N | mean | SD | N | mean | SD | N | mean | SD |
| Age (yr) | 63 | 68.55 | 11.73 | 4 | 76.75 | 6.39 | 67 | 69 | 11.23 |
| SBP (mmHg) | 63 | 135.79 | 15.94 | 4 | 137.5 | 13.02 | 67 | 136 | 15.3 |
| DBP (mmHg) | 63 | 77.14 | 10.66 | 4 | 71.75 | 7.41 | 67 | 77 | 10 |
| BMI (kg/m^2^) | 63 | 25.60 | 2.49 | 4 | 21.75 | 2.5 | 67 | 25.4 | 2.49 |
| HR (beats/min) | 63 | 65 | 8.07 | 4 | 67 | 7.09 | 67 | 66 | 8 |

HTN = hypertension, HCM = hypertrophic cardiomyopathy, CAD = coronary artery disease, yr = years, SBP = systolic blood pressure, DBP = diastolic blood pressure, BMI = body mass index, HR= heart rate, Gr = group, N = number, SD = standard deviation
