# Supplementary material for: A Novel Integrated Score Index of Echocardiographic Indices for the Evaluation of Left Ventricular Diastolic Function
Source: PLoS One. 2015 Nov 10;10(11):e0142175. doi: 10.1371/journal.pone.0142175 (PMC4640516; doi:10.1371/journal.pone.0142175)
Supplement: S3 File — (DOCX) [file pone.0142175.s003.docx]

**Supplemental Material 3:** Medications for all the subjects

1. Healthy subjects (393 cases): They came to the hospital to receive a health examination only; probably only a minority of female participants were receiving hormone (estrogen) therapy (< 3%)
2. Medication for hypertension (145 patients): no drug intake: 37 cases (25.5%); anti-hypertension drugs: 108 cases (74.5%)
   1. Calcium channel antagonist alone: 16%
   2. Angiotensin II antagonist alone: 13%
   3. Calcium channel antagonist + angiotensin II antagonist: 32%
   4. Calcium channel antagonist + β_1_-blocker: 4%
   5. Angiotensin II antagonist + β_1_-blocker: 23%
   6. Calcium channel antagonist + angiotensin II antagonist + β_1_-blocker: 10%
   7. β_1_-blocker alone: 4%
3. Medication for hypertrophic cardiomyopathy (24 cases)
   1. Calcium channel antagonists (nifedipine): 54%
   2. Spironolactone (short-term trial < 24 months): 25%
4. Medication for coronary artery disease (67 cases)
   1. Anti-platelets and thrombolytics (clopidogrel and/or aspirin): 97%
   2. Angiotensin II antagonists and/or calcium channel antagonists, and/or carvedilol for those with associated hypertension: 63%
   3. Anti-anginal agents: 45%
   4. Lipid lowering agents: 32%
